# Evaluating Post-Radiotherapy Laryngeal Function with Laryngeal Videostroboscopy in Early Stage Glottic Cancer

**DOI:** 10.3389/fonc.2017.00124

**Published:** 2017-06-12

**Authors:** Ariel E. Marciscano, Vivek Charu, Heather M. Starmer, Simon R. Best, Harry Quon, Alexander T. Hillel, Lee M. Akst, Ana P. Kiess

**Affiliations:** ^1^Department of Radiation Oncology and Molecular Radiation Sciences, The Johns Hopkins University School of Medicine, Baltimore, MD, United States; ^2^The Johns Hopkins University School of Medicine, Baltimore, MD, United States; ^3^Department of Otolaryngology (Head and Neck Surgery), Stanford University School of Medicine, Stanford, CA, United States; ^4^Department of Otolaryngology-Head and Neck Surgery, The Johns Hopkins University School of Medicine, Baltimore, MD, United States

**Keywords:** stroboscopy, laryngeal videostroboscopy, radiotherapy, glottic cancer, larynx cancer, dysphonia

## Abstract

**Objective:**

Dysphonia is common among patients with early stage glottic cancer. Laryngeal videostroboscopy (LVS) has not been routinely used to assess post-radiotherapy (RT) voice changes. We hypothesized that LVS would demonstrate improvement in laryngeal function after definitive RT for early-stage glottic cancer.

**Study design:**

Blinded retrospective review of perceptual voice and stroboscopic parameters for patients with early glottic cancer and controls.

**Setting:**

High-volume, single-institution academic medical center.

**Subjects and methods:**

Fifteen patients underwent RT for Tis-T2N0M0 glottic cancer and were evaluated with serial LVS exams pre- and post-RT. Stroboscopic assessment included six parameters: vocal fold (VF) vibration, VF mobility, erythema/edema, supraglottic compression, glottic closure, and secretions. Grade, roughness, breathiness, asthenia, strain (GRBAS) voice perceptual scale was graded in tandem with LVS score. Assessments were grouped by time interval from RT: pre-RT, 0–4, 4–12, and >12 months post-RT.

**Results:**

60 LVS exams and corresponding GRBAS assessments were reviewed. There were significant improvements in ipsilateral VF motion (*P* = 0.03) and vibration (*P* = 0.001) and significant worsening in contralateral VF motion (*P* < 0.001) and vibration (*P* = 0.008) at >12 months post-RT. Glottic closure significantly worsened, most prominent >12 months post-RT (*P* = 0.01). Composite GRBAS scores were significantly improved across all post-RT intervals.

**Conclusion:**

LVS proved to be a robust tool for assessing pre- and post-RT laryngeal function. We observed post-RT improvement in ipsilateral VF function, a decline in contralateral VF function, and decreased glottic closure. These results demonstrate that LVS can detect meaningful changes in VF and glottic function and support its use for post-RT evaluation of glottic cancer patients.

## Introduction

Voice preservation is a key consideration in treatment selection for patients with early stage glottic cancers. Definitive radiotherapy (RT) has been a mainstay in the management of this disease with excellent local control and survival rates (1–4). However, concerns remain regarding the late effects of RT including fibrosis, chronic edema, laryngeal stenosis, and xerostomia, all of which can impact voice outcomes and quality of life. The advent of endoscopic laser surgery (ELS) has introduced a relative shift in the treatment paradigm for this disease, as it offers comparable local control and laryngeal preservation rates to RT with limited morbidity and a range of therapeutic options for persistent and recurrent disease including repeat surgery and RT (5). Efforts have been made to compare voice outcomes between definitive RT and ELS, but results have been highly variable. Indeed some studies have reported equivalent outcomes (6–11) and others have reported superior results with either RT (12–14) or ELS (15, 16). There is a paucity of high-quality evidence directly comparing functional and perceptual voice outcomes between modalities. Furthermore, there is a lack of standardization in the evaluation of posttreatment dysphonia (17, 18).

Laryngeal videostroboscopy (LVS) is the gold standard for evaluation of dysphonia and laryngeal function (19, 20). LVS is an endoscopic tool that uses synchronized pulsed light at a frequency allowing the examiner to observe normal and pathologic vocal fold (VF) vibration and movement during phonation (21). Within the oncologic realm, LVS has most commonly been used for diagnosis of early stage glottic cancers but has also been employed as a metric of posttreatment voice. LVS offers improved diagnostic sensitivity over non-stroboscopic videolaryngoscopy as it permits evaluation of functional changes in VF biomechanics and vibration. LVS can be used in tandem with validated perceptual voice assessments in order to correlate dysphonia with specific physiologic findings. To date, there is limited experience using LVS to assess the impact of definitive RT upon functional voice outcomes and the underlying mechanisms of post-RT dysphonia remain poorly understood (16, 22, 23).

The objective of this work is to (1) evaluate changes over time in functional voice outcomes among patients with early stage glottic cancers treated with definitive RT and (2) to assess the robustness of LVS as a metric of post-RT dysphonia by analyzing inter-rater reliability between blinded expert reviewers.

## Materials and Methods

### Patient Selection

Patients with early stage glottic squamous cell carcinoma (Tis-T2N0M0) treated with definitive RT were screened for inclusion in a retrospective institutional review board-approved database. Eligibility criteria included patients with a pre-RT LVS exam and at least one post-RT LVS exam that underwent RT between 2009 and 2014. LVS exams and corresponding audio recordings were collected using an endoscope with distal chip technology and stroboscopic light source (Olympus ENF-V2 digital video rhinolaryngoscope; Olympus CLL-S1 StorobeLED Light Source, Center Valley, PA, USA) and stored on D-Scope^®^ Systems Workstation (D-Scope Systems, Brooklyn, NY, USA). A total of 15 patients were included in the study and 60 archived, de-identified videos were graded by four blinded reviewers, including two control exams. The expert reviewers included three fellowship-trained laryngologists (Alexander T. Hillel, Simon R. Best, and Lee M. Akst) and one speech-language pathologist (Heather M. Starmer) with experience in cancer-related dysphonia.

### Assessed Parameters

Patient information including sex, age at diagnosis, smoking history, and tumor stage per American Joint Committee on Cancer (AJCC seventh ed.) were recorded. Treatment characteristics of cumulative RT dose, RT dose per fraction, and number of fractions as well as RT technique [conventional or intensity modulated radiation therapy (IMRT)] were noted. For patients treated with IMRT, the clinical target volume encompassed the entire larynx with coverage of biltateral VF. In our practice, IMRT is utilized for a minority of patients, predominantly for carotid sparing among selected patients with a history of vascular disease or considered to be at high risk of vasculopathy due to medical comorbidities. Follow-up was calculated from the date of initiation of RT until the last LVS exam. Of note, six patients underwent definitive RT due to persistent disease after laser excision.

Patient assessments were grouped by temporal relationship to RT: (1) pre-RT (baseline), (2) 0–4 months (acute), (3) 4–12 months (subacute), and (4) >12 months (late). LVS exams were evaluated by six stroboscopic parameters: VF mobility, VF vibration, erythema/edema, supraglottic compression, glottic closure, and glottic secretion. The laterality of dysfunction was noted for VF motion and vibration assessments. The ipsilateral side was defined as the disease-involved VF and the contralateral side was defined as the uninvolved VF. For cases in which the lesion involved the anterior commissure and/or both VFs (*n* = 4), clinical judgment was used to define the ipsilateral side as the more involved VF. The severity of dysfunction for each LVS parameter was quantified using a scoring system ranging from 0 to 4, as follows: 0 = no dysfunction, 1 = minimal dysfunction, 2 = mild dysfunction, 3 = moderate dysfunction, 4 = severe dysfunction. Binary assessments were used for glottic closure (complete or incomplete) and glottic secretions (normal or thickened). An independent score for each parameter on all exams was determined by the blinded reviewer and then grouped by post-RT time interval in order to generate mean change over time (mean change = post-RT mean LVS score—pre-RT mean LVS score).

Perceptual voice evaluation was performed using the validated grade, roughness, breathiness, asthenia, strain (GRBAS) scale (24). A composite GRBAS score that ranged from 0 to 12 was generated from the sum of the individual variables using the following schema: 0 = no impairment, 1 = mild impairment, 2 = moderate impairment, 3 = severe impairment.

### Statistical Analysis

A paired Student’s *t*-test with its exact permutation distribution was used to assess change from baseline LVS score over time for each stroboscopic parameter. The Fisher’s exact test was used to assess categorical variables. Reported *p*-values are two-tailed, testing the null hypothesis that RT had no effect on LVS scores from baseline. A paired Student’s *t*-test was also used to assess change from the baseline composite GRBAS score.

To quantify agreement between expert reviewer LVS scores, the intra-class correlation coefficient (ICC) for each LVS parameter was determined at each time interval. The ICC is the correlation between measurements on the same patient exam by different reviewers (25). ICC values near 1.0 indicate perfect agreement and we considered ICC values of 0.81–1.0 as “very good,” 0.61–0.8 as “good,” 0.41–0.6 as “moderate,” 0.21–0.4 as “fair,” and ≤0.2 as “poor” correlation, respectively.

## Results

### Baseline Characteristics

The characteristics of the 15 patients included in the study are summarized in Table 1. The median age at diagnosis was 65 years (range, 44–87 years) and all patients were males. The majority of patients (60%) reported a smoking history with a median of 30 total pack-years (range, 10–60 pack-years). Two patients were active smokers during treatment. The distribution of tumor stage was; Tis (13%), T1a (33%), T1b (27%), T2 (27%).

**Table 1:** Baseline clinical and treatment characteristics for early stage glottic cancer patients (*n* = 15)^a^.

Baseline characteristics	Early stage glottic cancer patients
Total no. of patients	15
Gender	
Male	15 (100%)
Female	0 (0%)
Age at diagnosis, years	
Median (range)	65 (44–87)
Smoking history	
Never smoker	6 (40%)
Any smoking history	9 (60%)
Median (range), total pack years	30 (10–60)
Patient follow-up after RT, months	
Median (range)	20 (1–49)
Tumor (T) stage of glottic larynx cancer^b^	
Tis (carcinoma *in situ*)	2 (13%)
T1a	5 (33%)
T1b	4 (27%)
T2	4 (27%)
RT characteristics	
Median total dose (range), Gy	66 (63–70)
Median dose per fraction (range), Gy	2 (2–2.25)
Median no. of fractions (range)	33 (29–35)
Median elapsed time (range), days	44 (37–49)
RT technique	
Conventional RT	7 (47%)
IMRT	8 (53%)

*RT, radiation therapy; Gy, Gray; IMRT, intensity-modulated radiation therapy*.

*^a^Values are reported as number (%) of patients unless otherwise indicated*.

*^b^Based on American Joint Committee on Cancer TNM Staging System, seventh ed*.

The proportion of patients treated with conventional RT and IMRT was 47 and 53%, respectively. The median cumulative RT dose was 66 Gy (range 63–70 Gy) and the most common treatment schedules were 63 Gy in 28 fractions and 66 Gy in 33 fractions. The median duration of post-RT patient follow-up was 20 months (range, 1–49 months).

### Assessment of LVS Parameters

Each stroboscopic parameter was analyzed by the magnitude of change in LVS score from baseline to post-RT group. Given that increasing severity of dysfunction was denoted by higher LVS scores, a negative mean change in LVS score indicates post-RT improvement in dysfunction while a positive mean change indicates post-RT worsening (Table 2).

**Table 2:** Change in LVS parameters over time compared to pre-RT baseline function (*n* = 15).

LVS parameter	Pre-RT (baseline)	Acute (0–4 months post-RT)		Subacute (4–12 months post-RT)		Late (>12 months post-RT)	
	Mean score (±SEM)	Mean change (±SEM)	*P*-value	Mean change (±SEM)	*P*-value	Mean change (±SEM)	*P*-value
VF motion							
Ipsilateral	1.0 (±0.3)	−0.4 (±0.2)	0.1	−0.6 (±0.1)	0.01	−0.4 (±0.1)	0.03
Contralateral	0.3 (±0.1)	+0.1 (±0.1)	0.3	0 (±0.1)	0.8	+0.5 (±0.2)	<0.001
VFvibration							
Ipsilateral	3.2 (±0.2)	−0.4 (±0.3)	0.03	−0.9 (±0.2)	0.002	−0.9 (±0.3)	0.001
Contralateral	1.5 (±0.3)	−0.1 (±0.2)	0.5	−0.1 (±0.2)	0.7	+0.7 (±0.2)	0.008
Supraglottic compression	2.3 (±0.2)	+0.4 (±0.2)	0.01	+0.01 (±0.2)	0.9	+0.2 (±0.2)	0.3
Edema/erythema	1.4 (±0.2)	+0.7 (±0.2)	<0.001	+0.6 (±0.2)	0.002	+0.7 (±0.1)	0.002

	**% of patients (*n* = 15/59 exams)**	**% of patients (*n* = 11/41 exams)**	***P*-value**	**% of patients (*n* = 15/58 exams)**	***P*-value**	**% of patients (*n* = 15/60 exams)**	***P*-value**

Glottic closure^a^ (complete vs. incomplete)	88 vs. 12%	88 vs. 12%	1.0	90% vs. 10%	1.0	67 vs. 33%	0.01
Glottic secretiona^a^ (normal vs. thickened)	55 vs. 45%	25 vs. 75%	0.003	43% vs. 57%	0.3	38 vs. 62%	0.09

*LVS, laryngeal videostroboscopy; RT, radiation therapy; VF, vocal fold*.

*Pre-RT (baseline): 0 = no dysfunction, 1 = minimal dysfunction, 2 = mild dysfunction, 3 = moderate dysfunction, 4 = severe dysfunction. Post-RT groups are reported as mean change over time; mean change = post-RT Mean LVS score—pre-RT Mean LVS score*.

*^a^Fisher’s test used to assess categorical variables; paired Student’s t-test used to assess all other variables*.

### VF Motion and Vibration

**Figure 1:**
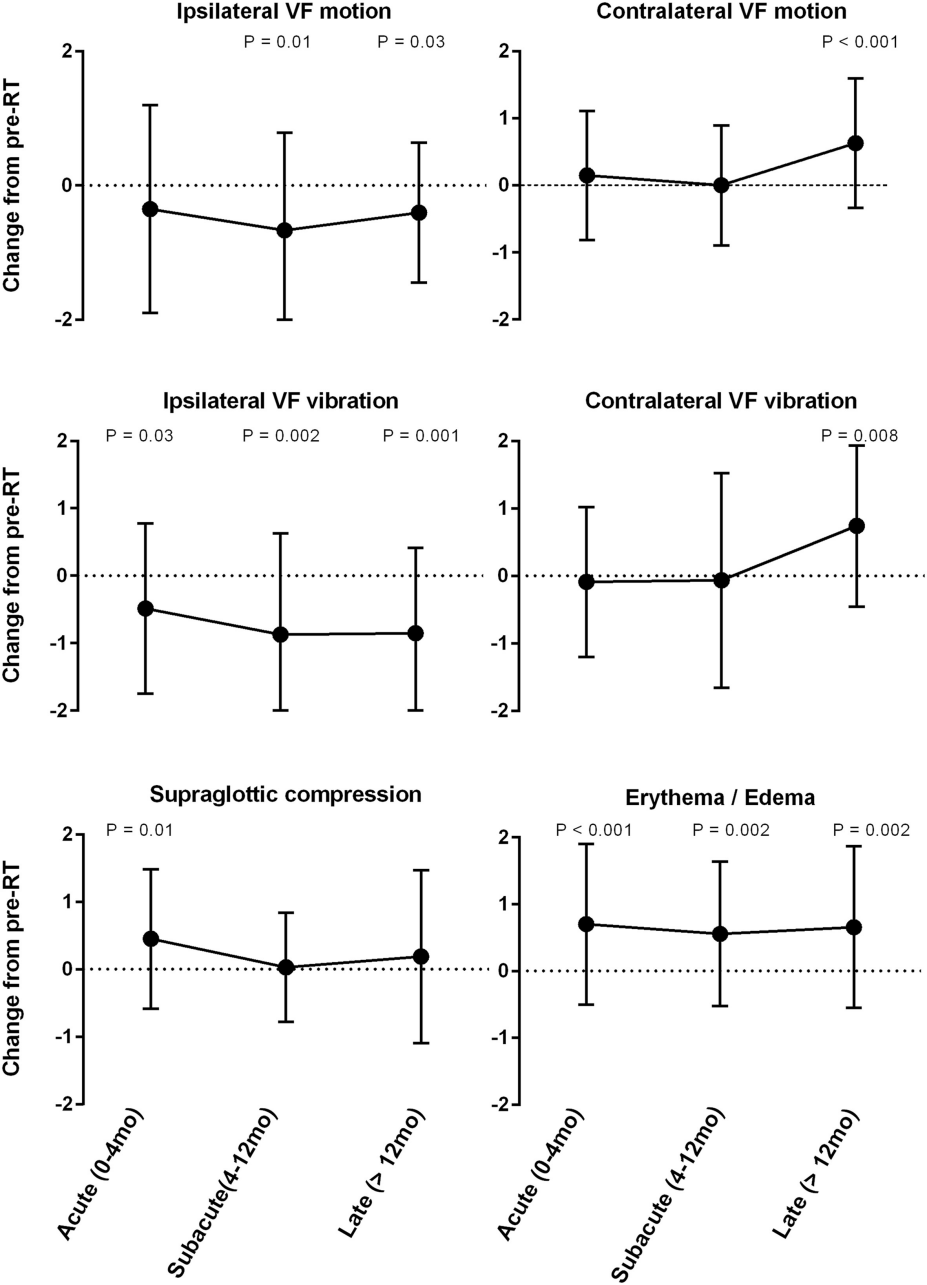
Mean change from pre-radiotherapy (RT) baseline score by laryngeal videostroboscopy parameter.

Stroboscopic assessment of ipsilateral and contralateral VF function yielded significant yet contrasting results (Figure 1). There was significant improvement in ipsilateral VF vibration observed across all post-RT time intervals. The magnitude of improvement in ipsilateral vibration was greater during the subacute (−0.9; *P* = 0.002) and late phases (−0.9; *P* = 0.001) in comparison to the acute phase (−0.4; *P* = 0.03). Ipsilateral VF motion demonstrated a gradual improvement over time with significantly less dysfunction during the subacute (−0.6; *P* = 0.01) and late phases (−0.4; *P* = 0.03). An unfavorable trend was observed with regards to contralateral VF function. Indeed, serial LVS demonstrated significant worsening of contralateral VF vibration (+0.7; *P* = 0.008) and motion (+0.8; *P* < 0.001) during the late post-RT phase.

Evaluation of VF vibration was unable to be performed in approximately 15% of cases reviewed by the blinded reviewers, which was consistent across all post-RT time intervals. The most common reason was limited visualization due to supraglottic compression and edema.

### Glottic Closure

**Figure 2:**
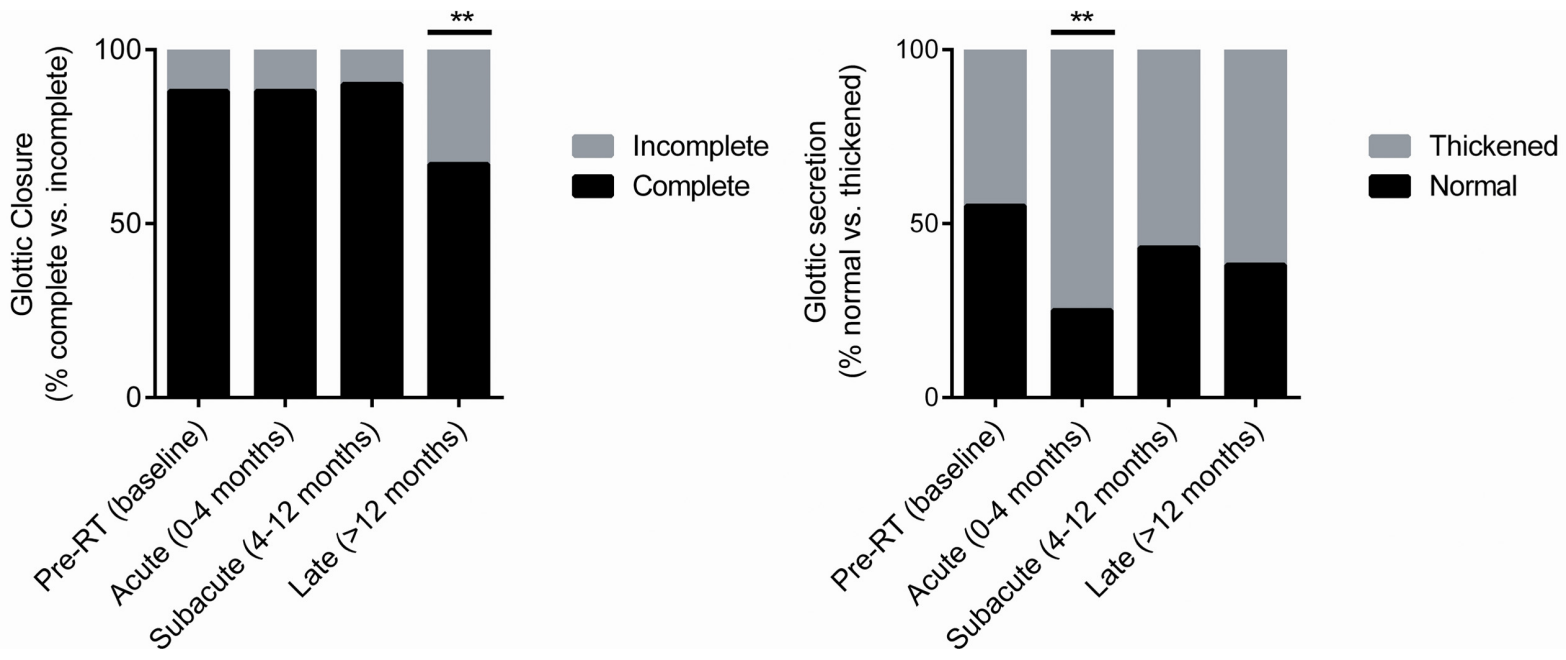
Pre-radiotherapy (RT) and post-RT change in the proportion of patients with complete glottic closure and normal glottic secretions over time.

In our series, approximately 88% (52 of 59; *n* = 15) of patients had complete glottic closure at baseline (Figure 2). The proportion of patients with complete glottic closure remained consistent during the acute (88%; 36 of 41, *n* = 11) and subacute (90%; 52 of 58, *n* = 15) post-RT phases. However, during the late post-RT phase, there was a significant decrease in the proportion of patients with complete glottic closure (67%; 40 of 60, *n* = 15; *P* = 0.01). These results suggest a decline in function due to adverse treatment effect such as radiation-induced fibrosis, a well characterized late complication following RT (26–29).

### Glottic Secretions

A significant (*P* = 0.003) increase in the proportion of patients with thickened secretions during the acute post-RT phase (75%, 33 of 44, *n* = 11) was noted (Figure 2). In comparison to baseline, in which 45% of patients had thickened secretions, we did not observe statistically meaningful changes during the subacute and late posttreatment intervals. We postulate that acute phase changes relate to acute radiation mucositis that resolves as the mucosal injury heals. It should be noted that there was a potential trend (*P* = 0.09) toward thickened secretions in the late post-RT interval. It is plausible that this finding is driven by alternative mechanisms such as salivary gland dysfunction and/or xerostomia.

### Supraglottic Compression and Laryngeal Erythema/Edema

Similar to the trends observed with glottic secretions, there was a significant increase in the severity of supraglottic compression during the acute post-RT phase (+0.4; *P* = 0.01), which did not persist during later phases. Additionally, laryngeal edema/erythema were significantly increased across all post-RT intervals (*P* ≤ 0.002) and demonstrated the greatest mean change from baseline among all LVS parameters. These findings are expected as both edema and erythema are common sequela following irradiation.

### GRBAS Composite Score

Grade, roughness, breathiness, asthenia, strain composite score was used to correlate voice quality with LVS parameters. Across all post-RT intervals, there was a significant improvement in voice impairment (Table 3). The magnitude of improvement was greatest during the late phase (−3.9; *P* < 0.001), suggesting gradual improvement in voice quality. Importantly, despite a decrease in contralateral VF motion and vibration, particularly during the late phase, perceptual voice quality was consistently better than pre-RT baseline.

**Table 3:** Change in grade, roughness, breathiness, asthenia, strain (GRBAS) composite score over time compared to pre-RT baseline function (*n* = 15)^a^.

GRBAS parameters	Pre-RT (baseline)	Acute (0–4 months post-RT)		Subacute (4–12 months post-RT)		Late (>12 months post-RT)	
	Mean score (±SEM)	Mean change (±SEM)	*P*-value	Mean change (±SEM)	*P*-value	Mean change (±SEM)	*P*-value
GRBAS composite	(±0.9)	−3.0 (±0.8)	0.004	−1.4 (±0.7)	0.1	−3.9 (±0.6)	<0.001

*RT, radiation therapy*.

*^a^Paired Student’s t-test used to assess GRBAS composite score*.

### Inter-Rater Reliability

Given the paucity of experience utilizing LVS to assess post-RT functional outcomes and the inherent subjectivity of this tool, we sought to evaluate inter-rater reliability between blinded reviewers using ICC (Table 4). Encouragingly, the majority of LVS parameters demonstrated good correlation during at least one time interval. The exceptions were contralateral VF motion and laryngeal edema/erythema, which had moderate correlation as their highest mark of inter-rater reliability. Overall, ipsilateral VF vibration demonstrated the most consistency among all LVS parameters while contralateral VF motion was the least reliable parameter, particularly during the acute and subacute phases. When analyzing by LVS parameter, we observed the following trend in order of decreasing ICC: ipsilateral VF vibration, supraglottic compression, ipsilateral VF motion, contralateral VF vibration, laryngeal edema/erythema, and contralateral VF motion. With respect to consistency across time intervals, there was a general trend of declining ICC over time, although the subacute group demonstrated the lowest inter-rater reliability. In summary, LVS demonstrated a reasonable degree of inter-rater reliability when assessing RT-related changes over time. Importantly, ipsilateral VF motion and vibration were among the most consistent LVS parameters in our series, and this may have clinical implications from the standpoint of surveillance for post-RT local recurrence.

**Table 4:** Evaluation of interrater reliability by ICC for LVS parameters (*n* = 15).

LVS parameter	Pre-RT (baseline)	Acute (0–4 months post-RT)	Subacute (4–12 months post-RT)	Late (>12 months post-RT)
	ICC (95% CI)	ICC (95% CI)	ICC (95% CI)	ICC (95% CI)
VF motion				
Ipsilateral	0.80 (0.7–0.9)	0.64 (0.4–0.9)	0.35 (−0.1–0.8)	0.49 (0.1–0.9)
Contralateral	0.53 (0.2–0.9)	0.30 (−0.1–0.7)	0.24 (−0.1–0.5)	0.51 (0.1–0.9)
VF vibration				
Ipsilateral	0.66 (0.4–0.9)	0.76 (0.6–0.9)	0.49 (0.1–0.8)	0.61 (0.3–0.9)
Contralateral	0.70 (0.6–0.8)	0.48 (0.1–0.8)	0.54 (0.1–1.0)	0.33 (−0.2–1.0)
Supraglottic compression	0.54 (0.2–0.9)	0.80 (0.7–0.9)	0.60 (0.3–0.9)	0.49 (0.2–0.8)
Edema/erythema	0.37 (0.01–0.7)	0.43 (−0.01–0.9)	0.60 (0.4–0.8)	0.58 (0.3–0.8)

*ICC, intra-class correlation coefficient; LVS, laryngeal videostroboscopy; RT, radiation therapy; CI, confidence interval; VF, vocal fold*.

## Discussion

A majority of patients with early stage glottic cancer treated with either definitive RT or surgery are cured (5). Therefore, therapy selection is largely driven by the ability to achieve functional organ preservation and to reduce side effects without compromising tumor control. RT and minimally invasive ELS have emerged as two viable options for management of early stage glottic cancer given comparable oncologic outcomes with minimal associated morbidity in comparison to open surgery. ELS and RT result in different structural and functional changes within the larynx; however, it remains unclear, which approach provides superior voice outcomes and which factors should guide therapy selection. As such, LVS may serve as a clinically useful tool to improve understanding of posttreatment voice dysfunction in early stage glottic cancer. Furthermore, adoption of LVS as a pre- and post-RT surveillance tool may provide opportunity for early detection and intervention to reduce long-term treatment effects and optimize voice outcomes.

From a practical standpoint, each modality impacts the glottis and surrounding laryngeal anatomy in vastly different manners. Definitive RT is generally performed using a treatment field that encompasses the entire larynx, thus exposing the bilateral VC and adjacent normal tissues to high doses of radiation. As such, both acute and late toxicities often arise due to the irradiation of non-diseased tissues. It is generally accepted that post-RT dysphonia results from collateral damage to the contralateral VC and adjacent larynx as there is often notable improvement in the lesioned VC following definitive RT. On the other hand, the intent of ELS is to precisely, yet, completely remove the glottic lesion while minimizing disruption to the adjacent VC and surrounding laryngeal tissues. Therefore, the injury to the non-involved contralateral VC that occurs with RT is generally circumvented with ELS. The structural defect in the VC that results from endoscopic resection can cause functional perturbations, which lead to dysphonia. Indeed, the glottal gap on phonation impacts voice outcomes among post-ELS patients and is associated with the extent of cordectomy beyond the VF (30). Additionally, more invasive lesions or lesions extending into the anterior commissure have been correlated with inferior local control rates for patients undergoing ELS. Therefore, ELS is often selected for more superficial lesions while definitive RT may be preferred for early stage patients with less favorable disease characteristics (31–33). It is clear that ELS and RT cause different structural and functional changes within the larynx, thus providing rationale for the use of LVS to improve our understanding of posttreatment dysphonia in early stage glottic cancer.

This study reports our experience with LVS in order to characterize the pre- and posttreatment changes in laryngeal function in response to RT. The strengths of this work include: (1) detailed analysis of multiple videostroboscopic parameters and their modulation over time and relative to pretreatment baseline, (2) acquisition of GRBAS scores in tandem with LVS for correlative analysis of perceptual and functional voice outcomes, and (3) a panel of blinded expert reviewers provided unbiased assessments that permitted inter-rater reliability analysis of LVS in the post-RT setting. Ultimately, our aim was to understand the changes in laryngeal function in order to provide a mechanistic understanding of the post-RT improvement and/or decline in voice. In our series, we observed significant improvements in ipsilateral VF vibration and motion over time. These findings correspond with favorable tumor response and are indicative of restoration and/or improvement in function from pre-RT baseline. They also correlated with favorable perceptual voice outcomes as the composite GRBAS score demonstrated significant improvement in both acute and late post-RT phases. Interestingly, we observed a significant decline in contralateral VF function and glottic closure during the late post-RT phase. These findings suggest the development of late adverse treatment effect such as soft tissue fibrosis and/or laryngeal stenosis, which can impair phonation. Taken together, ipsilateral functional improvement and late contralateral functional decline provide a plausible mechanism for RT-mediated treatment effect and dysphonia. It merits note that non-stroboscopic endoscopy is able to assess several of the parameters evaluated by LVS in our series, however, our most clinically significant findings relate to changes in VF vibration, which requires stroboscopic assessment.

Along these lines, we also investigated whether more conformal RT techniques influenced functional voice outcomes in comparison to those treated with conventional RT techniques. We did not appreciate differences among the patients treated with IMRT vs. those treated with non-IMRT technique. These findings are expected as both techniques use treatment fields that encompass the entire larynx, including the bilateral VCs, and thus there is no meaningful reduction in dose to the uninvolved, contralateral laryngeal tissue. This is consistent with other reports, which have not identified improvement in voice outcomes. Alternatively, IMRT planning has been shown to reduce the severity of acute radiation dermatitis and to spare dose to the carotid vessels (34, 35). Intriguingly, preliminary reports from a prospective study examining the feasibility of IMRT-based single vocal cord irradiation for T1a glottic tumors has demonstrated promising 2-year local control rates and a modest, transient decline in voice quality by voice handicap index. These findings illustrate the potential to improve voice outcomes by minimizing irradiation of the contralateral VC (36–39).

Few studies have used LVS to evaluate post-RT functional outcomes and even fewer series have used LVS as a metric to compare outcomes between ELS and RT (10, 13, 16, 23, 40–42). A small Japanese series evaluated 10 patients with T1N0M0 glottic cancer with pre- and post-RT LVS (22). Rigorous analysis of stroboscopic parameters was not performed; however, there was a general trend toward improvement of mucosal waves following RT. McGuirt and colleagues retrospectively analyzed stroboscopic outcomes following irradiation or ELS among patients with T1a glottic cancers (10). Meaningful analysis was restricted by small sample size with LVS performed in only five patients who underwent RT. All patients treated with RT had complete glottic closure as compared with 80% in the ELS group and irradiated patients had abnormal mucosal waves in bilateral VFs while ELS patients demonstrated abnormal vibratory patterns only within the incised VF. Sjörgren et al. retrospectively assessed voice outcomes in 18 patients with T1a mid-cord glottic carcinoma treated with ELS and compared them with a historical cohort of 14 patients treated with definitive RT (40). LVS demonstrated abnormal patterns in the majority of patients regardless of treatment modality, and there were no differences between treatment groups. Among the patients treated with RT, 57% had incomplete glottic closure, 86% had VF asymmetry, and 29% had abnormal vibratory patterns. With regards to mucosal wave assessment, nine patients (64%) had some abnormality of which four patients had bilateral reduced or absent mucosal waves. Finally, a Dutch group retrospectively evaluated vocal function in a series of T1N0M0 glottic cancer patients treated with RT (16). Forty-five patients underwent perceptual voice analysis and LVS assessment over multiple post-RT time points. Prior to RT, the majority of patients had abnormalities of mucosal wave, vibration, or glottic closure and the proportion of patients with normal exams following RT ranged 60–86%, suggesting functional improvement. Our work represents a significant contribution to the existing body of literature; we have comprehensively analyzed six videostroboscopic parameters and their modulation over multiple clinically relevant time points and, importantly, compared them with pre-RT baseline function.

We acknowledge the limitations associated with our single institution retrospective analysis. The limited cohort size restricted our statistical analysis, and we were underpowered to further assess clinical and treatment-related factors that may influence LVS parameters. Additionally, the lack of a validated grading schema to assess functional outcomes with LVS and the inter-rater heterogeneity are unique challenges encountered in our study. Finally, the heterogeneity of dose-fractionation schedules and RT techniques utilized introduce additional variables, which may complicate interpretation of our findings.

## Conclusion

Laryngeal videostroboscopy is a robust tool for assessing pre- and post-RT functional voice outcomes among patients with early-stage glottic cancer with reasonable inter-rater reliability. We observed significant post-RT improvement in ipsilateral VF function corresponding to favorable treatment response and overall improvement in perceptual voice quality. We also observed a late decline in contralateral VF function and glottic closure. Our work provides a foundation for future prospective validation of LVS among patients undergoing definitive RT.

## Ethics Statement

This is a retrospective single-institution series, all patients included were part of an institutional review board/IRB-approved database.

## Author Contributions

Research concept/design: AM, HS, SB, HQ, AH, LA, and AK. Analysis: AM, VC, HS, SB, HQ, AH, LA, and AK. Author VC is Ph.D. trained biostatistician. Manuscript writing/preparation: AM, VC, HS, SB, AH, LA, and AK.

## Conflict of Interest Statement

The author LA serves Laryngology Advisory Board for Olympus America, Inc. The author AH has research support from the NIH/NIDCD and has provided expert witness testimony in medical legal cases. The terms of this arrangement have been reviewed and approved by The Johns Hopkins University in accordance with its policy on objectivity in research.

## Abbreviations

LVS, laryngeal videostroboscopy; RT, radiotherapy; VF, vocal fold; ICC, intra-class correlation coefficient; ELS, endoscopic laser surgery; Gy, gray; IMRT, intensity-modulated radiation therapy.
